# Supplementary material for: Implantable cardioverter-defibrillator therapy after resuscitation from cardiac arrest in vasospastic angina: A retrospective study
Source: PLoS One. 2022 Oct 31;17(10):e0277034. doi: 10.1371/journal.pone.0277034 (PMC9621437; doi:10.1371/journal.pone.0277034)
Supplement: S1 Table — Data are shown as the number (%). Ach, acetylcholine; CAG, coronary angiography; ECG, electrocardiogram; LAD, left anterior descending artery; LCX, left circumflex artery; RCA, right coronary artery; VSA, vasospastic angina. (DOC) [file pone.0277034.s001.doc]

**Table S1. Diagnostic methods for VSA used for patients with VSA in this study**

| Variable | VSA  (n=51) |
| --- | --- |
| Diagnostic methods of VSA |  |
| ACh provocation test | 37 (73%) |
| Spontaneous ST-elevation | 13 (25%) |
| Severe spasm on emergent CAG | 1 (2%) |
| Details of ACh provocation test |  |
| Site of induced spasm |  |
| LAD | 26/37 (80%) |
| RCA | 23/37 (62%) |
| LCX | 14/37 (38%) |
| Multivessel spasm | 18/37 (49%) |
| ST-segment elevation on ECG | 22/37 (59%) |
| ST-segment depression on ECG | 9/37 (24%) |
| Chest pain | 29/37 (80%) |

Data are shown as the number (%).

Ach, acetylcholine; CAG, coronary angiography; ECG, electrocardiogram; LAD, left anterior descending artery; LCX, left circumflex artery; RCA, right coronary artery; VSA, vasospastic angina
