# Supplementary material for: Implantable cardioverter-defibrillator therapy after resuscitation from cardiac arrest in vasospastic angina: A retrospective study
Source: PLoS One. 2022 Oct 31;17(10):e0277034. doi: 10.1371/journal.pone.0277034 (PMC9621437; doi:10.1371/journal.pone.0277034)
Supplement: S2 Table — Data are shown as mean ± standard deviation, median [interquartile range], or number (%). * Data for current smoking were missing in 22 patients. ATP, anti-tachycardia pacing; BNP, brain natriuretic peptide; eGFR, estimated glomerular filtration rate; ICD, implantable cardioverter-defibrillator; ICM, ischemic cardiomyopathy; LVEF, left ventricular ejection fraction; PEA, pulseless electrical activity; SCA, sudden cardiac arrest; VF, ventricular fibrillation; VSA, vasospastic angina; VT, ventricular tachycardia. (DOC) [file pone.0277034.s002.doc]

**Table S2. Patient characteristics among etiologies**

| Variables | VSA  (n = 51) | ICM  (n = 86) | Non-ICM  (n = 42) | Brugada  Syndrome  (n = 16) | Long QT  Syndrome  (n = 12) | Sarcoidosis  (n = 13) | Others  (n = 60) | *P*-values |
| --- | --- | --- | --- | --- | --- | --- | --- | --- |
| Age (years) | 54.6 ± 11.7 | 66.7 ± 11.6 | 65.7 ± 12.0 | 39.8 ± 15.4 | 52.9 ± 14.1 | 60.4 ± 10.5 | 57.4 ± 15.8 | <0.001 |
| Male | 38 (75%) | 72 (84%) | 32 (76%) | 16 (100%) | 4 (33%) | 10 (77%) | 47 (78%) | 0.002 |
| Body mass index (kg/m^2^) | 23.1 ± 3.9 | 23.8 ± 3.2 | 23.5 ± 4.2 | 22.5 ± 2.7 | 26.9 ± 7.7 | 22.9 ± 3.3 | 22.8 ± 4.0 | 0.242 |
| Hypertension | 26 (51%) | 63 (73%) | 23 (55%) | 3 (19%) | 4 (33%) | 5 (38%) | 30 (50%) | <0.001 |
| Diabetes mellitus | 7 (14%) | 34 (40%) | 13 (31%) | 0 (0%) | 2 (17%) | 7 (54%) | 8 (13%) | <0.001 |
| Dyslipidaemia | 16 (31%) | 61 (71%) | 12 (29%) | 1 (6%) | 2 (17%) | 5 (38%) | 12 (20%) | <0.001 |
| Current smoker* | 14/45 (31%) | 17/74 (23%) | 3/42  (7%) | 1/12  (8%) | 1/11  (9%) | 1/13  (8%) | 6/55  (11%) | 0.024 |
| Haemoglobin (g/dl) | 13.4 ± 2.2 | 12.9 ± 2.0 | 13.0 ± 1.9 | 14.1 ± 1.2 | 11.5 ± 2.3 | 13.3 ± 2.0 | 12.8 ± 2.0 | 0.022 |
| eGFR (ml/min/1.73 m^2^) | 72.3 ± 19.0 | 56.3 ± 22.3 | 55.9 ± 15.2 | 84.4 ± 19.8 | 74.7 ± 20.2 | 69.4 ± 22.5 | 72.0 ± 30.1 | <0.001 |
| BNP (pg/ml) | 53 [15–91] | 187 [89–431] | 346 [100–619] | 15 [9–24] | 26 [13–189] | 134 [66–379] | 87 [32–187] | <0.001 |
| LVEF (%) | 64.7 ± 7.8 | 40.1 ± 13.3 | 51.7 ± 16.7 | 69.3 ± 8.5 | 62.9 ± 7.8 | 41.4 ± 9.1 | 51.6 ± 13.1 | <0.001 |
| Family history of SCA | 0 (0%) | 2 (2%) | 3 (7%) | 0 (0%) | 3 (25%) | 0 (0%) | 3 (5%) | 0.022 |
| Initial rhythm of SCA |  |  |  |  |  |  |  | 0.522 |
| VT/VF | 49 (96%) | 85 (99%) | 42 (100%) | 16 (100%) | 12 (100%) | 12 (92.3%) | 59 (98%) |  |
| PEA/Asystole | 2 (4%) | 1 (1%) | 0 (0%) | 0 (0%) | 0 (0%) | 1 (8%) | 1 (2%) |  |
| Follow-up duration (years) | 4.1 [2.0–6.9] | 3.4 [2.1–5.0] | 3.2 [2.0–5.8] | 5.3 [4.0–6.9] | 6.1 [1.6–7.4] | 4.0 [2.3–4.9] | 3.8 [2.0–6.9] | 0.103 |
| Composite endpoint | 12 (24%) | 24 (28%) | 15 (36%) | 7 (44%) | 2 (17%) | 4 (31%) | 23 (38%) | 0.411 |
| All-cause death | 2 (4%) | 7 (8%) | 5 (12%) | 0 (0%) | 1 (8%) | 3 (23%) | 6 (10%) | 0.253 |
| Cardiac death | 1 (2%) | 3 (3%) | 3 (7%) | 0 (0%) | 0 (0%) | 2 (15%) | 2 (3%) | 0.361 |
| Appropriate ICD therapy | 11 (22%) | 19 (22%) | 13 (31%) | 7 (44%) | 1 (8%) | 2 (15%) | 19 (32%) | 0.214 |
| ATP only | 4 (8%) | 8 (9%) | 5 (12%) | 1 (6%) | 1 (8%) | 0 (0%) | 6 (10%) | 0.785 |
| Shock | 7 (14%) | 11 (13%) | 8 (19%) | 6 (38%) | 0 (0%) | 2 (15%) | 13 (22%) | 0.093 |
| Inappropriate ICD therapy | 5 (10%) | 8 (9%) | 4 (10%) | 1 (6%) | 0 (0%) | 1 (8%) | 4 (7%) | 0.841 |
| Infection/lead disconnection | 2 (4%) | 0 (0%) | 1 (2%) | 0 (0%) | 1 (8%) | 1 (8%) | 0 (0%) | 0.129 |

Data are shown as mean ± standard deviation, median [interquartile range], or number (%). * Data for current smoking were missing in 22 patients. ATP, anti-tachycardia pacing; BNP, brain natriuretic peptide; eGFR, estimated glomerular filtration rate; ICD, implantable cardioverter-defibrillator; ICM, Ischaemic cardiomyopathy; LVEF, left ventricular ejection fraction; PEA, pulseless electrical activity; SCA, sudden cardiac arrest; VF, ventricular fibrillation; VSA, vasospastic angina; VT, ventricular tachycardia.
