# Supplementary material for: Implantable cardioverter-defibrillator therapy after resuscitation from cardiac arrest in vasospastic angina: A retrospective study
Source: PLoS One. 2022 Oct 31;17(10):e0277034. doi: 10.1371/journal.pone.0277034 (PMC9621437; doi:10.1371/journal.pone.0277034)
Supplement: S3 Table — Chronic kidney disease was defined as glomerular filtration rate <60 mL/min/1.73 m2. ACE-I, angiotensin converting enzyme inhibitor; ARB, angiotensin receptor blockers; CI, confidence interval; HR, hazard ratio; SCA, sudden cardiac arrest; VSA, vasospastic angina. (DOC) [file pone.0277034.s003.doc]

**Table S3. Factors associated with death and recurrent ventricular arrhythmias in patients with VSA**

| Variables | Univariable analysis | | |  | Multivariable analysis | | |
| --- | --- | --- | --- | --- | --- | --- | --- |
|  | HR | *P*-values | 95% CI |  | HR | *P*-values | 95% CI |
| Age (years) | 1.01 | 0.792 | 0.95–1.06 |  |  |  |  |
| Male | 0.49 | 0.227 | 0.16–1.56 |  |  |  |  |
| Body mass index (kg/m^2^) | 0.95 | 0.493 | 0.80–1.11 |  |  |  |  |
| Hypertension | 0.29 | 0.062 | 0.08–1.06 |  | 0.36 | 0.131 | 0.09-1.36 |
| Dyslipidaemia | 0.91 | 0.880 | 0.27–3.03 |  |  |  |  |
| Diabetes mellitus | 1.47 | 0.618 | 0.32–6.75 |  |  |  |  |
| Smoking history | 2.55 | 0.265 | 0.49-13.17 |  |  |  |  |
| Chronic kidney disease | 4.10 | 0.017 | 1.28–13.11 |  | 3.24 | 0.052 | 0.99–10.58 |
| Left ventricular ejection fraction (%) | 1.50 | 0.758 | 0.94–1.09 |  |  |  |  |
| Medication |  |  |  |  |  |  |  |
| Calcium channel blocker | 0.36 | 0.184 | 0.08-1.63 |  |  |  |  |
| Long-acting nitrate | 1.20 | 0.764 | 0.36-4.00 |  |  |  |  |
| Nicorandil | 1.01 | 0.986 | 0.32-3.19 |  |  |  |  |
| ACE-I or ARB | N/A | 0.999 | N/A |  |  |  |  |
| β-blocker | N/A | 0.999 | N/A |  |  |  |  |
| Amiodarone | 1.23 | 0.842 | 0.16-9.63 |  |  |  |  |
| Statin | 0.49 | 0.497 | 0.06-3.81 |  |  |  |  |

Chronic kidney disease was defined as glomerular filtration rate < 60 mL/min/1.73 m^2^.

ACE-I, angiotensin converting enzyme inhibitor; ARB, angiotensin receptor blockers; CI, confidence interval; HR, hazard ratio; SCA, sudden cardiac arrest; VSA, vasospastic angina.
