# Supplementary material for: Implantable cardioverter-defibrillator therapy after resuscitation from cardiac arrest in vasospastic angina: A retrospective study
Source: PLoS One. 2022 Oct 31;17(10):e0277034. doi: 10.1371/journal.pone.0277034 (PMC9621437; doi:10.1371/journal.pone.0277034)
Supplement: S4 Table — Data are shown as mean ± standard deviation, median (interquartile range), or number (%). * Data for current smoking are missing in 7 patients. ACE-I, angiotensin converting enzyme inhibitor; ACh: acetylcholine; ARB, angiotensin receptor blockers; BNP: brain natriuretic peptide; CAG, coronary angiography; ECG, electrocardiogram; eGFR: estimated glomerular filtration rate; ICD, implantable cardioverter defibrillator; LAD, left anterior descending artery; LCX, left circumflex artery; LVEF: left ventricular ejection fraction; PEA: pulseless electrical activity; RCA, right coronary artery; SCA: sudden cardiac arrest; VF: ventricular fibrillation; VSA: vasospastic angina; VT: ventricular tachycardia. (DOCX) [file pone.0277034.s004.docx]

**Table S4. Characteristics of patients with and without ICD in VSA and SCA**

| Variable | All patients  (n=75) | ICD (+)  (n=51) | ICD (−)  (n=24) | P value |
| --- | --- | --- | --- | --- |
| Age (years) | 57.3±12.2 | 54.6±11.7 | 62.9±11.3 | 0.005 |
| Male | 56 (75%) | 38 (75%) | 18 (75%) | 1.000 |
| Body mass index (kg/m^2^) | 22.7±3.6 | 23.1±3.9 | 21.9±2.7 | 0.240 |
| Hypertension | 40 (53%) | 26 (51%) | 14 (58%) | 0.624 |
| Diabetes mellitus | 10 (13%) | 7 (14%) | 3 (13%) | 1.000 |
| Dyslipidemia | 23 (31%) | 16 (31%) | 7 (29%) | 1.000 |
| Current smoker* | 20/68 (29%) | 14/45 (31%) | 6/23 (26%) | 0.782 |
| Hemoglobin (g/dl) | 13.2±2.0 | 13.4±2.2 | 12.8±1.5 | 0.259 |
| eGFR (ml/min/1.73 m^2^) | 66.1±22.1 | 72.3±19.0 | 53.0±22.9 | <0.001 |
| BNP (pg/ml) | 47 [17-93] | 53 [15-91] | 40 [18-127] | 0.095 |
| LVEF (%) | 64.0±9.2 | 64.7±7.8 | 62.2±11.8 | 0.288 |
| Family history of SCA | 1 (1%) | 0 (0%) | 1 (4%) | 0.320 |
| Initial rhythm of SCA |  |  |  | 0.078 |
| VT/VF | 69 (92%) | 49 (96%) | 20 (83%) |  |
| PEA/Asystole | 6 (8%) | 2 (4%) | 4 (17%) |  |
| Diagnostic methods of VSA |  |  |  |  |
| ACh provocation test | 46 (62%) | 37 (73%) | 9 (39%) | 0.005 |
| Spontaneous ST-elevation | 24 (32%) | 13 (25%) | 11 (46%) | 0.082 |
| Severe spasm on emergent CAG | 5 (7%) | 1 (2%) | 4 (17%) | 0.034 |
| Details of ACh provocation test |  |  |  |  |
| Site of induced spasm |  |  |  |  |
| LAD | 31/46 (67%) | 26/37 (80%) | 5/9 (56%) | 0.445 |
| RCA | 28/46 (61%) | 23/37 (62%) | 5/9 (56%) | 0.721 |
| LCX | 15/46 (33%) | 14/37 (38%) | 1/9 (11%) | 0.235 |
| Multivessel spasm | 19/46 (41%) | 18/37 (49%) | 1/9 (11%) | 0.061 |
| ST-segment elevation on ECG | 27/46 (59%) | 22/37 (59%) | 5/9 (56%) | 1.000 |
| ST-segment depression on ECG | 13/46 (28%) | 9/37 (24%) | 4/9 (44%) | 0.246 |
| Chest pain | 37/46 (80%) | 29/37 (80%) | 8/9 (89%) | 0.664 |
| Medication |  |  |  |  |
| Calcium channel blocker | 70 (93%) | 48 (95%) | 22 (92%) | 0.653 |
| Long-acting nitrate | 41 (55%) | 31 (61%) | 10 (42%) | 0.121 |
| Nicorandil | 43 (57%) | 29 (57%) | 14 (58%) | 0.904 |
| ACE-I or ARB | 13 (17%) | 5 (10%) | 8 (33%) | 0.020 |
| β-blocker | 7 (9%) | 4 (8%) | 3 (13%) | 0.673 |
| Amiodarone | 7 (9%) | 5 (10%) | 2 (8%) | 1.000 |
| Statin | 15 (20%) | 7 (14%) | 8 (33%) | 0.065 |
| Follow up duration | 3.8 [1.6-5.8] | 4.1 [2.0-6.9] | 2.7 [0.2-5.1] | 0.059 |
| All-cause death | 6 (8%) | 2 (4%) | 4 (17%) | 0.078 |
| Cardiac death | 5 (7%) | 1 (2%) | 4 (17%) | 0.034 |

Data are shown as mean ± standard deviation, median (interquartile range), or number (%). * Data for current smoking are missing in 7 patients.

ACE-I, angiotensin converting enzyme inhibitor; ACh: acetylcholine; ARB, angiotensin receptor blockers; BNP: brain natriuretic peptide; CAG, coronary angiography; ECG, electrocardiogram; eGFR: estimated glomerular filtration rate; ICD, implantable cardioverter defibrillator; LAD, left anterior descending artery; LCX, left circumflex artery; LVEF: left ventricular ejection fraction; PEA: pulseless electrical activity; RCA, right coronary artery; SCA: sudden cardiac arrest; VF: ventricular fibrillation; VSA: vasospastic angina; VT: ventricular tachycardia.
