# Supplementary figures and images for: Implantable cardioverter-defibrillator therapy after resuscitation from cardiac arrest in vasospastic angina: A retrospective study
Source: PLoS One. 2022 Oct 31;17(10):e0277034. doi: 10.1371/journal.pone.0277034 (PMC9621437; doi:10.1371/journal.pone.0277034)

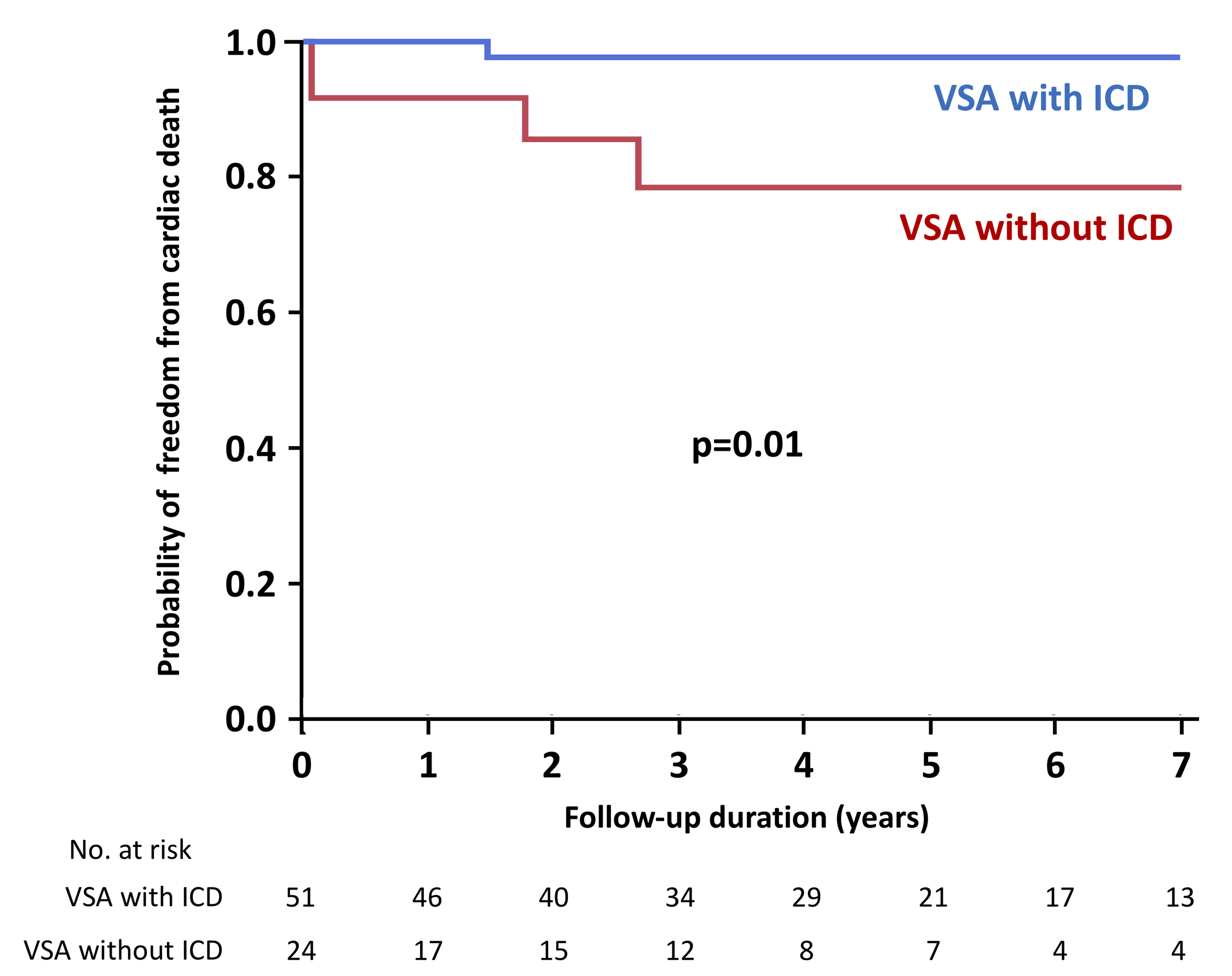

Supplement: S1 Fig — ICD, implantable cardioverter-defibrillator; SCA, sudden cardiac arrest; VSA, vasospastic angina. (TIF) [file pone.0277034.s005.tif]
